# Beneficial Alteration in Growth Performance, Immune Status, and Intestinal Microbiota by Supplementation of Activated Charcoal-Herb Extractum Complex in Broilers

**DOI:** 10.3389/fmicb.2022.856634

**Published:** 2022-04-15

**Authors:** Lixue Wang, Ying Zhang, Xiangyue Guo, Limin Gong, Bing Dong

**Affiliations:** State Key Laboratory of Animal Nutrition, College of Animal Science and Technology, China Agricultural University, Beijing, China

**Keywords:** activated charcoal-herb extractum complex, broiler, immune, growth performance, microbiota activated charcoal-herb extractum complex, microbiota

## Abstract

This study aimed to examine the effects of activated charcoal-herb extractum complex (CHC) on the growth performance of broilers, inflammatory status, microbiota, and their relationships. A total of 864 1-day-old Arbor Acres male broilers (41.83 ± 0.64 g) were distributed to eight dietary treatments with six replicates (18 birds per replicate), which were a corn-soybean meal-based diet (NCON); basal diets supplemented with 250, 500, 750, or 1,000 mg/kg CHC, and three positive controls; basal diets supplemented with 200 mg/kg antibacterial peptide (AMP), 200 mg/kg calsporin (Probio) or 500 mg/kg montmorillonite. The study period was 42 days including the starter (day 0–21) and grower (day 22–42) phases. Compared with the NCON group, CHC supplementation (optimal dose of 500 mg/kg) increased (*p* < 0.05) growth performance and tended to increase feed conversion rate in broilers. CHC (optimal dose of 500 mg/kg) decreased the level of the interleukin-1β (IL-1β) and interferon-γ (IFN-γ) in serum and improved the levels of immunoglobulins A (IgA) and immunoglobulins A (IgM) in serum, and secretory immunoglobulin A (SIgA) in the mucosa of duodenum and jejunum (*p* < 0.05). In the ileum, CHC supplementation decreased community abundance represented by lower Sobs, Chao 1, Ace, and Shannon compared with NCON (*p* < 0.05). At the phylum level, CHC supplementation increased the abundance of Firmicutes, while decreasing the abundance of Bacteroidetes in ileum and cecum (*p* < 0.05). At the genus level, compared with the NCON group, CHC markedly reduced (*p* < 0.05) the abundances of pathogenic bacteria *Alistipes* in the ileum, which were negatively associated with the levels of SIgA and IL-1β in ileum mucosa. In conclusion, CHC had beneficial effects on growth performance, immune status, and intestinal microbiota composition. CHC had dual functions of absorption like clays and antibacterial like antibacterial peptides.

## Introduction

Various factors related to diet, infectious disease pathogens, and environment have a negative impact on the delicate balance among components of the chicken gut and subsequently impair growth rate and feed conversion efficiency (Hughes, 2005). In addition, toxins cause disease when fed to broilers in contaminated feedstuffs (Ochieng et al., 2021). Feedstuffs and feed can be contaminated with toxins in farms, post-harvesting, or during storage (Patriarca and Fernández Pinto, 2017). Over the past eight decades, antibiotics have been used in poultry production to increase productivity and efficiency (Mehdi et al., 2018). However, scientific evidence suggests that the continuous use of antibiotics in feed and/or water has led to the development of antibiotic resistance in pathogenic bacteria (Forgetta et al., 2012), and the presence of antibiotic residues in animal products and the environment (Gonzalez Ronquillo and Angeles Hernandez, 2017). These outcomes of continuous antibiotic use can compromise human and animal health (Diarra et al., 2010). Therefore, there is an urgent need to find effective additives that are both benefit growth performance and weaken gut harmful bacteria.

Activated charcoal-herb extractum complex (CHC) is produced by active charcoal’s sorption of extractum from Chinese herbs. Consequently, CHC possesses dual functions of absorption and antibacterial properties (Wang et al., 2019). Activated charcoal has been proven to absorb mycotoxins in feed (Jindal et al., 1994; Burchacka et al., 2019; Santos and van Eerden, 2021) and improve the growth performance of broilers (Oso et al., 2014). Activated charcoal could clear the immune suppression induced by mycotoxins (Khatoon et al., 2018; Bhatti et al., 2021). Chinese herbs (such as *Pulsatilla chinensis*, *Portulaca oleracea L.*, *Artemisia argyi Folium*, and *Pteris multifida Poir*) have been used widely in China clinically for the treatment of digestive infections including enteritis, bacillary dysentery, and intestinal amoebiasis owing to its anti-bacterial, anti-inflammatory, and immunomodulatory activities (Wang and Wang, 2010; Liu et al., 2011; Wang and Lv, 2016; Iranshahy et al., 2017; Lan et al., 2020). CHC can reduce diarrhea in weaned pigs (Wang et al., 2019), but the effects of CHC on growth performance, immune response, and intestinal microbial composition in broilers have not been investigated.

Charcoal-herb extractum complex has two primary properties of interest. First, CHC contains activated charcoal that metabolizes or absorbs toxins in the gastrointestinal tract and prevents absorption and entrance of toxins to the liver (Zellner et al., 2019). Second, Chinese medicines are effective treatments for various infectious diseases (Wang and Yang, 2011). However, during the production of CHC, cross-reactions among active radicals may negatively affect the functionality of the activated charcoal and Chinese medicines. Thus, we selected three antibiotic alternatives with clear functional mechanisms as positive controls to compare to CHC activities. First, montmorillonite (MMT) is widely used as a feed additive due to its capacity to bind mycotoxins in animal feed and the gastrointestinal tract (Liu et al., 2021). Second, an antibacterial peptide (AMP) is a prebiotic product that contains secondary metabolites of *Bacillus licheniformis* and has strong inhibitory effects on gram-positive bacteria (Chen et al., 2021). Finally, *B. subtilis* strain (Probio) can favor the growth of beneficial microbiota and lactic acid-producing bacteria (Alakomi et al., 2000; Hoa et al., 2000).

In this study, we examined the effects of CHC on the growth performance of broilers and the potential effects of CHC on the absorption of toxins and antibacterial effects in the gastrointestinal tract, and the related immune status of broilers.

## Materials and Methods

This experiment was carried out at the National Feed Engineering Technology Research Center at the Ministry of Agriculture Feed Industry Center Animal Testing Base (Hebei, China). All procedures here were conducted in accordance with the Chinese Guidelines for Animal Welfare and approved by the Laboratory Animal Welfare and Animal Experimental Ethical Inspection Committee of China Agricultural University (AW503800710-2-2).

### Preparation of Charcoal-Herb Extractum Complex

An activated charcoal-herb extractum complex was obtained from the Fujian Baicaoshaung Biotechnology Co., Ltd. (Nanping, China). The manufacture of CHC was reported in a previous paper (Wang et al., 2019). In brief, CHC is formed by active charcoal sorption of Chinese herbs extractum. Analysis of elemental composition demonstrated that approximately 90% of CHC was carbon. Activated carbon was prepared by crushing and screening cedarwood and pine wood, followed by carbonization and activation at high temperatures (120–130°C). After that, the activated carbon was screened and only the part with the size range of 0.18 to 0.25 mm was retained for the next step. The mixture of traditional Chinese herbs included a raw plant of *Pulsatilla chinensis* (dried rhizome), *Portulaca oleracea L.* (dried whole plant), *Artemisia argyi Folium* (dried whole plant), and *Pteris multifida Poir* (dried whole plant) in the ratio of 7:8:10:6. The plant mixture was washed and soaked in the five-folds volume of water for 12 h. Subsequently, the plant mixture was decocted twice at 100°C under normal pressure, each for 1 h, then the decoction was filtered and collected, followed by concentration under a low pressure to a small volume (∼0.2-fold volume of initial plant volume) of Chinese herbs extractum. Finally, Chinese herb extractum were mixed with activated carbon in a proportion of 1:9 for 8 h and then dried to make CHC product. AMP was purchased from Qilu Pharmaceutical Co., Ltd. (Shandong, China). Probio (*B. subtilis* strain C-3102, Calsporin) was purchased from Shanghai Muguan Enterprise Development Co., Ltd. (Shanghai, China). MMT was purchased from Guangzhou Jingmu Feed Co., Ltd. (Guangzhou, China).

### Experiment Animals, Design, and Diets

Arbor Acres male broilers (*n* = 864; one day of age; initial BW = 41.83 ± 0.64 g) were allocated randomly to eight treatment groups, with six replicate pens per treatment and 18 broiler chickens per pen. Dietary treatments were: (1) negative control: corn-soybean meal-based diet (NCON), (2) NCON with 200 mg/kg Antibacterial peptide (AMP), (3) NCON with 200 mg/kg calsporin (Probio, equivalent to adding 2.0 × 10^9^ CFU/g of viable *B. subtilis*), (4) NCON with 500 mg/kg montmorillonite (MMT), and (5) NCON with 250, 500, 750, or 1,000 mg/kg CHC. The optimal supplemental level was selected according to the growth performance in CHC treatments compared to NCON. CHC treatment with optimal dose was used as representative CHC group to analyze intestinal microbial composition with the controls.

The 42-day experimental period included a starter phase (day 0–21) and a grower phase (day 22–42). The basal diet was formulated to meet or exceed estimates of the nutrient requirements of broilers (Table 1) suggested by the (National Research Council [NRC], 1994). Prior to the mixing process, the feed ingredients have been weighed and ground. The ingredients, composed less than 1% (w/w) of the diet (vitamins, minerals, and CHC), were mixed well with soybean meal in advance. Finally, all ingredients were all put into a feed blender and mixed well. The broiler shed contained equipment for ventilation, heating, and lighting. Every wire-mesh cage (90 cm × 90 cm × 75 cm) housed six chicks, each equipped with two automatic teat waterers and a round feeder (diameter: 37 cm). A lighting program with 24-h continuous light was used throughout the trial. All broiler chickens had *ad libitum* access to feed and water. Environmental temperature for 1-day-old broilers was controlled to about 33°C and then gradually decreased to 24°C at week 3 (the temperature was lowered to 3°C every week), which was maintained at this temperature thereafter. Broilers were vaccinated with Newcastle disease vaccine on day 7 and vaccinated with an inactivated infectious bursal disease vaccine on day 14.

**TABLE 1:** Composition and nutrient concentration of basal diets (%, as-fed basis).

Item	Starter (0–21 day)	Grower (22–42 day)
**Ingredients,%**		
Corn	59.85	60.80
Soybean meal	30.13	28.63
Fish meal	4.00	2.77
Soybean oil	2.75	4.54
Dicalcium phosphate	0.88	1.00
Limestone	1.45	1.38
DL-Methionine, 98%	0.14	0.08
Salt	0.30	0.30
Vitamin-mineral premix^a^	0.50	0.50
Total	100.00	100.00
**Nutrient concentration^b^**		
Digestible energy, kcal/kg	3,050	3,150
Crude protein	21.46	20.00
Calcium	1.00	0.95
Total phosphorus	0.68	0.67
Methionine	0.50	0.41
Lysine	1.13	1.04
**Mycotoxin level, measured^c^**		
DON, μg/kg	1128.17	1082.96
ZEA, μg/kg	255.81	240.34
AFB1, μg/kg	3.72	4.29

*^a^Premix provided the following per kg of feed: vitamin A, 10,000 IU; vitamin D3, 3,000 IU; vitamin E, 24 mg; vitamin K_3_, 2.1 mg; vitamin B_12_, 2 mg; riboflavin, 5.0 mg; pantothenic acid, 15 mg; niacin, 40 mg; choline chloride, 500 mg; folic acid, 0.9 mg; vitamin B_6_, 3.0 mg; biotin, 0.05 mg; Mn (from MnSO_4_⋅H_2_O), 70 mg; Fe (from FeSO_4_⋅H_2_O), 80 mg; Zn (from ZnSO_4_⋅H_2_O), 100 mg; Cu (from CuSO_4_⋅5 H_2_O), 18.8 mg; I (from KI), 0.35 mg; Se (from Na_2_SeO_3_), 0.30 mg. ^b^Values were calculated according to NRC (1994). ^c^Values were measured by UPLC-MS/MS analysis. AFB1, aflatoxin B1; DON, deoxynivalenol; ZEA, zearalenone.*

### Sample Collection and Processing

The body weight of birds was recorded on days 1, 21, and 42 of the experiment after 12 h withdrawing of feed but not water. Body weight gain (BWG) was calculated for 1–21, 21–42, and 1–42 days periods. Feed intake (FI) was recorded for the same periods and feed conversion ratio (FCR) was calculated after correcting for mortalities. Two phases of feed samples were collected, and stored at −20°C prior to mycotoxin analysis.

On days 21 and 42, one broiler closest to the average BW of each pen was selected for blood sampling. Blood samples were collected from the jugular vein. Serum was collected after centrifugation at 1,300 × *g* for 10 min at 4°C and stored (−20°C) for analysis of immune and inflammatory indices. On day 42, after blood collection, birds from each treatment (*n* = 6) were weighed and then killed by cervical decapitation.

On day 42, the small intestine was divided into three parts: the duodenum (from the pylorus to the ligament of Treitz), the jejunum (from the distal portion of the duodenal loop to Meckel’s diverticulum), and the ileum (anterior portion of the ileocecal junction). The intestine was sliced open lengthwise and gently rinsed in ice-cold PBS. Mucosa from the mid-duodenum, mid-jejunum, and mid-ileum of birds from each treatment (*n* = 6) was scraped with a glass slide. The glass slide with scrapings was rapidly placed in liquid nitrogen for measurement of intestinal immune and inflammatory indices. Gut contents from the ileum, cecum, and colon of birds from each treatment (*n* = 5) were collected into sterile plastic bags and immediately chilled on dry ice for analysis of intestinal microbiota.

### Mycotoxin Content

Mycotoxin content was measured using ultra-high-performance liquid chromatography-tandem mass spectrometry (UPLC-MS/MS) analysis. Briefly, the feed sample was extracted according to the QuEChERS method with slightly modified (Oplatowska-Stachowiak et al., 2015). The analysis was performed using the Acquity UPLC I-class system (Waters, Milford, MA, United States) and mass spectrometer (Xevo TQ-S, Waters) according to Kolawole et al. (2020).

### Inflammatory and Immune Status

Serum immunoglobulins A, M, and G (IgA, IgM, and IgG) were measured with an automatic biochemical analyzer (Hitachi 7600, Hitachi High-Technologies Corporation, Tokyo, Japan), following the instructions that accompanied commercially available kits for each immunoglobulin (Nanjing Jiancheng Bioengineering Institute, Nanjing, China). Concentrations of interleukin-1β (IL-1β) and interferon-γ (IFN-γ) in serum and mucosa of duodenum, jejunum, and ileum were determined by enzyme-linked immunosorbent assay (ELISA) kits (Nanjing Jiancheng Bioengineering Institute, Nanjing, China). Intestinal secretory immunoglobulin A (SIgA) concentrations were assayed using an Sn-69513-type immune counter (Shanghai Nucleus Annular Photoelectric Instrument Co., Ltd., Shanghai, China).

### Microbiota Analysis

Microbial DNA was extracted from ileal, cecal, and colonic digesta using the E.Z.N.A.^®^ stool DNA kit (Omega Bio-Tek, Norcross, GA, United States) according to the manufacturer’s instructions. Markers and adaptor-linked universal primers 338F (ACTCCTACGGGAGGCAGCAG) and 806R (GGACTACHVGGGTWTCTAAT) targeting the V3-V4 region were used to amplify microbial 16S rRNA. The PCR amplification of the 16S rRNA gene was performed as follows: 95°C for 3 min, followed by 25 cycles at 95°C for 30 s, 55°C for 30 s, and 72°C for 45 s and a final extension at 72°C for 10 min. The 2% agarose gel was used for the detection of purity of PCR products, and the products were purified with the AxyPrep DNA Gel Extraction kit (Axygen Biosciences, Union City, CA, United States). Purified amplicons were pooled in equimolar proportions and paired-end sequenced (2 × 300) on an Illumina MiSeq platform (Illumina, San Diego, CA, United States). The processing of sequencing data was conducted as previously described (Wang et al., 2021). Sequenced raw reads were deposited into the NCBI sequence read archive database (Accession Number: PRJNA794039).

After demultiplexing, the resulting sequences were merged with FLASH (v 1.2.11) (Magoč and Salzberg, 2011) and quality filtered with fastp (v 0.19.6) (Chen et al., 2018). Then the high-quality sequences were de-noised using DADA2 (Callahan et al., 2016) plugin in the QIIME 2 (version 2020.2) pipeline with recommended parameters, which obtains single nucleotide resolution based on error profiles within samples (Bolyen et al., 2019). DADA2 denoised sequences are usually called amplicon sequence variants (ASVs). ASVs were aligned with MAFFT and used to construct a phylogeny with FastTree2. Taxonomic assignment of ASVs was performed using the Naive bayes consensus taxonomy classifier implemented in QIIME 2 and the SILVA 16S rRNA database (v138). Analyses of the 16S rRNA microbiome sequencing data was performed using the free online platform of Majorbio Cloud Platform (cloud.majorbio.com).

### Statistical Measurements

#### Growth Performance and Immunological Parameters Analysis

The GLM procedure of SAS (SAS Institute Inc., Cary, NC, United States) was used for data analysis. Each pen served as an experimental unit for broiler growth performance. Individual broiler was considered the experimental unit for inflammation and immune status data. Duncan’s multiple comparisons were used for analyzing differences between groups. Coefficients for unequally spaced contrasts were generated by the interactive matrix algebra procedure (IML) of SAS. Then, orthogonal polynomial contrast was applied to assess the linear and quadratic responses of CHC. Differences were regarded statistically significant at *p* < 0.05, and 0.05 ≤ *p* < 0.10 was indicative of a differential trend.

#### Microbiota Analysis

The α-diversity indices, including the Shannon diversity index (Shannon), ACE estimator (Ace), Chao 1 estimator (Chao 1), and observed richness (Sobs) were expressed as mean and compared among NCON, CHC, AMP, Probio, and MMT. For α-diversity, the Kruskal–Wallis test was used for comparing multiple groups, and Mann–Whitney *U* test was used for two groups. Differences between β-diversity indices were determined by the ANOSIM test. Principal coordinates analysis (PCoA) plots were generated using the “ggplot2” packages of the R software (version 3.3.1). Also, we performed Permutational Multivariate Analysis of Variance (PERMANOVA) (Anderson, 2001) using 999 permutations based on Bray–Curtis distances using the Adonis function in the package “vegan” in R software (version 3.3.1). The significant differential bacteria at phylum and genus level were determined by using analysis of the composition of microbiomes (ANCOM). *p* < 0.05 was considered statistically significant for the overall effect, and false discovery rate (FDR) < 0.10 was significant for individual contrasts in order to incorporate a stringent false discovery rate (Feye et al., 2019). Linear discriminant analysis (LDA) effect size (LEfSe) (Segata et al., 2011) was conducted to find differences in the relative abundance of bacteria among treatments. LEfSe scores were used to measure the consistency of differences in relative abundance between the taxa analyzed in the groups (NCON vs. CHC vs. AMP vs. Probio vs. MMT). The higher score, the higher consistency. The significant taxa were set at LDA score > 3.5 and *p* < 0.05. Spearman correlation coefficient among bacterial taxa and the concentrations of immune indices and inflammatory cytokines were calculated using the “Hmisc” packages of the R software (version 3.3.1), and the visualization work was done using “pheatmap” packages of R software. Functions of microbial community from ileal, cecal, and colonic digesta were predicted by Reconstruction of Unobserved States (PICRUSt2) analysis. Significant differences in microbiological communities among groups were evaluated by ANOSIM with the R package, “vegan.”

## Results

### Growth Performance

During the starter phase, compared with the NCON group, supplementation of 500 mg/kg CHC in diets increased BWG (*p* < 0.01, Table 2). As the level of CHC in the diet increased, BWG tended to increase linearly (*p* = 0.058) and responded quadratically (*p* = 0.030). Compared with the AMP and Probio groups, dietary supplementation of 500 mg/kg CHC increased BWG (*p* < 0.01), and tended to improve FCR (*p* = 0.062). There was no difference in growth performance among broilers fed positive controls and those fed 0, 250, 750, or 1,000 mg/kg CHC (*p* > 0.05).

**TABLE 2:** Effects of CHC on the growth performance of broilers (*n* = 6)^1^.

Item	AMP	Probio	MMT	CHC, mg/kg					SEM^2^	*p* value		
				0	250	500	750	1,000		ANOVA^3^	Linear^4^	Quadratic^5^
**1–21 day**												
BWG, g	307.6^b^	325.3^b^	329.7^ab^	323.5^b^	330.3^ab^	370.1^a^	342.9^ab^	345.0^ab^	9.80	<0.01	0.058	0.03
FI, g	492.1	563.2	578.1	499.8	496.6	552.3	539.3	520.7	27.41	0.219	0.322	0.323
FCR	1.6	1.74	1.76	1.55	1.49	1.49	1.57	1.50	0.073	0.062	0.929	0.801
**22–42 day**												
BWG, g	1106.7	1063.6	1088.4	995.5	1108.2	1204.3	1048.3	1029.3	43.75	0.067	0.952	<0.01
FI, g	1992.1	1999.5	1959.1	1927.2	1896.7	1963.8	1863.7	1838.0	48.95	0.206	0.037	0.18
FCR	1.81	1.90	1.80	1.98	1.72	1.64	1.78	1.80	0.074	0.089	0.196	<0.01
**1–42 day**												
BWG, g	1414.3^ab^	1388.8^ab^	1418.1^ab^	1319.1^b^	1438.4^ab^	1574.4^a^	1391.2^ab^	1374.3^ab^	45.36	0.024	0.62	<0.01
FI, g	2463.5	2492.7	2490.9	2457.1	2423.6	2549.9	2435.7	2386.0	52.42	0.510	0.312	0.079
FCR	1.75	1.81	1.76	1.88	1.69	1.62	1.75	1.74	0.054	0.081	0.196	<0.01

*^1^Experimental diets were a corn-soybean meal basal diets (NCON) and basal diets supplemented with 250, 500, 750, or 1,000 mg/kg activated charcoal-herb extractum complex (CHC). Three additional diets containing 200 mg/kg antibacterial peptide (AMP), 200 mg/kg calsporin (Probio) or basal diets with 500 mg/kg montmorillonite (MMT) were set as the positive controls. ^2^Values listed for means and pooled SEM of all data are actual data. ^3^Statistical significance was determined using one-way ANOVA among all groups (including the three positive controls). ^4,5^Linear and quadratic effects of added CHC levels, except for the positive controls (AMP, Probio, MMT). ^a,b^Means within rows with different letter superscripts differ (p < 0.05). BWG average daily gain, FI average daily feed intake, FCR feed to gain ratio.*

During the grower phase, growth performance was not affected by CHC treatment. Compared with AMP, Probio, and MMT groups, feeding 500 mg/kg CHC tended to improve BWG (*p* = 0.067) and FCR (*p* = 0.089) of broilers.

Over the whole experimental period, BWG and FCR quadratically responded with an increasing level of CHC (*p* < 0.01). Compared with the NCON group, dietary CHC supplementation at a dosage of 500 mg/kg increased BWG (*p* = 0.024), while other doses showed no effect (*p* > 0.05). In addition, supplementation of 500 mg/kg CHC tended to improve FCR (*p* = 0.081), compared with three positive controls.

### Serum Immunological Parameters

Concentrations of serum IgA (*p* < 0.01) on day 21 when feeding 500 mg/kg and 750 mg/kg CHC were higher than that in NCON (Table 3). Concentrations of serum IgA (*p* < 0.01) on day 42 and IgM (*p* = 0.018) on day 21 at the level of 500 mg/kg CHC was higher than in the negative control. On day 21 and day 42, serum IgA tended to increase linearly with increased level of CHC from 250 mg/kg to 1,000 mg/kg (*p* = 0.079 and *p* = 0.091, respectively). The concentrations of serum IgA (day 21 and day 42) and IgM (day 21) showed quadratic (*p* < 0.01) responses to increased CHC levels. Serum IgG (*p* > 0.05) was not affected by CHC on both day 21 and day 42. Immune responses in the 500 mg/kg CHC group were similar to those observed in the three positive controls. Compared with the three positive controls, supplementation of CHC at 500 mg/kg increased serum concentrations of IgA (*p* < 0.01) both on day 21 and day 42.

**TABLE 3:** Effects of CHC on serum immunological parameters in broilers (*n* = 6)^1^.

Item	AMP	Probio	MMT	CHC, mg/kg					SEM^2^	*P* value		
				0	250	500	750	1000		ANOVA^3^	Linear^4^	Quadratic^5^
**21 day**												
IgA, g/L	1.07^bc^	1.08^bc^	0.97^bc^	0.77^c^	0.98^bc^	1.44^a^	1.11^ab^	0.92^bc^	0.074	<0.01	0.079	<0.01
IgG, g/L	5.74	7.23	6.41	5.94	6.39	8.04	6.93	6.35	0.664	0.292	0.54	0.08
IgM, g/L	0.69^b^	0.68^b^	0.78^ab^	0.69^b^	0.81^ab^	1.06^a^	0.84^ab^	0.70^ab^	0.082	0.018	0.757	<0.01
IL-1β, pg/ml	32.08^a^	29.27^abc^	26.28^bcd^	29.37^ab^	27.60^bcd^	25.22^d^	26.35^cd^	25.69^cd^	0.803	<0.01	<0.01	0.045
IFN-γ, pg/ml	38.43^ab^	39.00^ab^	27.79^cd^	43.85^a^	28.30^cd^	24.09^d^	33.18^bc^	32.90^bc^	1.616	<0.01	<0.01	<0.01
**42 day**												
IgA, g/L	1.04^bc^	1.00^c^	1.10^bc^	1.09^bc^	1.15^bc^	1.37^a^	1.19^b^	1.19^b^	0.088	<0.01	0.091	<0.01
IgG, g/L	7.62	6.95	7.79	7.58	9.48	9.65	9.18	8.2	0.908	0.318	0.736	0.066
IgM, g/L	0.93	0.84	0.99	0.94	0.82	0.97	1.02	0.82	0.113	0.824	0.899	0.384
IL-1β, pg/ml	30.20^abc^	31.71^ab^	30.81^abc^	32.88^a^	28.62^bcd^	25.92^d^	28.47^bcd^	30.05^abc^	0.707	<0.01	0.033	<0.01
IFN-γ, pg/ml	39.00^ab^	34.61^b^	38.43^ab^	40.45^a^	34.73^b^	34.19^b^	34.04^b^	39.99^a^	1.622	0.017	0.803	<0.01

*^1^Experimental diet were corn-soybean meal basal diets (NCON) and basal diets supplemented with 250, 500, 750, or 1,000 mg/kg CHC. Three additional diets containing 200 mg/kg antibacterial peptide (AMP), 200 mg/kg calsporin (Probio), or basal diets with 500 mg/kg montmorillonite (MMT) were set as the positive controls. ^2^Values listed for means and pooled SEM of all data are actual data. ^3^Statistical significance was determined using one-way ANOVA among all groups (including the three positive controls). ^4,5^Linear and quadratic effects of added CHC levels, except for the positive controls (AMP, Probio, MMT). ^a–d^Means within rows with different letter superscripts differ (p < 0.05). IgA immunoglobulin A, IgG immunoglobulin G, IgM immunoglobulin M, IL-1β interleukin-1β, IFN-γ interferon γ.*

Concentrations of serum IL-1β (day 21 and day 42) and IFN-γ (day 21) linearly (*p* < 0.01) decreased with increasing concentrations of CHC and responded quadratically (*p* < 0.05) on both day 21 and day 42. Dietary supplementation of 500 mg/kg CHC decreased levels of IL-1β (*p* < 0.01) and IFN-γ (*p* < 0.05) in the serum of broilers compared with NCON. Broilers fed 500, 750, and 1,000 mg/kg CHC displayed reduced serum concentrations of IL-1β (*p* < 0.01) and IFN-γ (*p* < 0.01) on day 21 compared with NCON. Similarly, 500 and 750 mg/kg CHC groups had lower concentrations of IL-1β (*p* < 0.01) and IFN-γ (*p* = 0.017) on day 42 comparing with NCON. Supplementation with 500 mg/kg CHC was more efficient in reducing serum IL-1β (*p* < 0.01) than AMP and Probio on day 21, and more efficient (*p* < 0.01) than positive controls on day 42. Supplementation with 500 mg/kg of CHC reduced IFN-γ (*p* < 0.01) more than AMP and Probio on day 21, while there was no difference between CHC and positive controls on day 42 (*p* > 0.05).

### Intestinal Immunological Parameters

**FIGURE 1:**
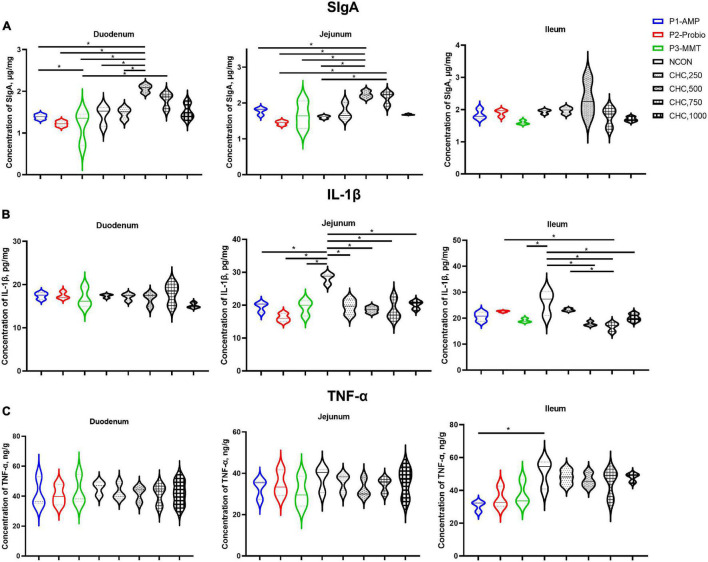
Effects of CHC on intestinal mucosa secretory immunoglobulin A (SIgA), interleukin-1β (IL-1β), and tumor necrosis factor-α (TNF-α) levels in broilers (*n* = 6). **(A)** The concentration of secretory immunoglobulin A (SIgA) in the mucosa of duodenum, jejunum, and ileum. **(B)** The concentration of interleukin-1β (IL-1β) in the mucosa of duodenum, jejunum, and ileum. **(C)** The concentration of tumor necrosis factor-α (TNF-α) in the mucosa of duodenum, jejunum, and ileum. The “*” indicates a significant difference between the two treatments (*p* < 0.05). Values are expressed as means (the solid line between two dashed lines) ± SEM. Experimental diets were corn-soybean meal basal diets (NCON) and basal diets supplemented with 250, 500, 750, or 1,000 mg/kg CHC. Three additional diets containing 200 mg/kg antibacterial peptide (AMP), 200 mg/kg calsporin (Probio), or basal diets with 500 mg/kg montmorillonite (MMT) were set as the positive controls.

Supplementation with 500 mg/kg CHC increased concentrations of SIgA in the mucosa of the duodenum and jejunum (*p* < 0.05) on day 42 compared to that of NCON. CHC at 750 mg/kg increased jejunum mucosa SIgA (*p* < 0.05) concentration compared with NCON (Figure 1). Duodenum and jejunum mucosa SIgA concentrations were higher (*p* < 0.05) in the broilers fed 500 mg/kg CHC compared with those in positive controls.

Concentrations of IL-1β in the mucosa of jejunum and ileum in the treatments of 500, 750, and 1,000 mg/kg CHC were lower (*p* < 0.05) than that in NCON on day 42. No significant differences were observed in the mucosa of duodenal IL-1β and in the mucosa of duodenal, jejunal, and ileal TNF-α concentrations among all treatments (*p* > 0.05). Broilers fed with 750 mg/kg CHC had lower levels of IL-1β (*p* < 0.05) in ileum mucosa when compared with the Probio group. Moreover, there was no difference between CHC and positive controls in the mucosa of the duodenum and jejunum (*p* > 0.05). Furthermore, the level of TNF-α (*p* > 0.05) in the mucosa of duodenum, jejunum, and ileum did not differ among CHC and positive controls.

### Microbial Diversity and Composition in Digesta

**FIGURE 2:**
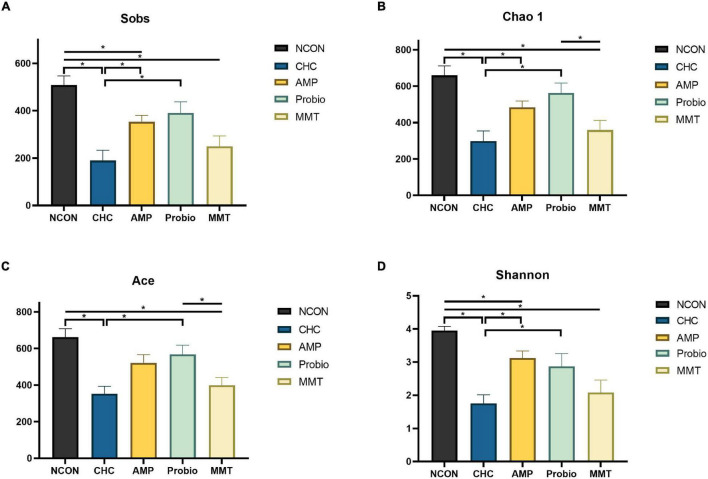
Effects of CHC on α-diversity of ileal microbiota in broilers on day 42. **(A)** Represents Sobs index. **(B)** Represents Chao 1 estimator. **(C)** Represents ACE estimator. **(D)** Represents Shannon index. Four indexes analyzed the α diversity of broilers from different directions. NCON is the corn-soybean meal basal diet group. AMP is a 200 mg/kg antibacterial peptide supplemented group. Probio is 200 mg/kg calsporin supplemented group. MMT is 500 mg/kg montmorillonite supplemented group. CHC is 500 mg/kg activated charcoal-herb extractum complex supplemented group. The Kruskal-Wallis test was used for comparing multiple groups, and Mann–Whitney *U* test was used for two groups. The “*” indicates *p* < 0.05.

**FIGURE 3:**
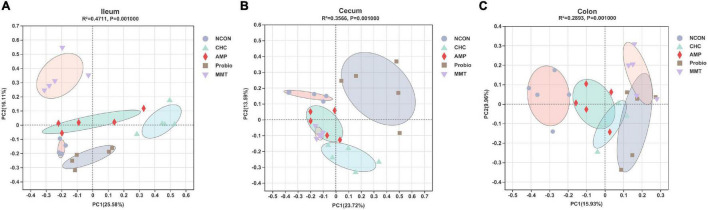
Principal coordinates analysis (PCoA) of bacterial communities in the intestinal digesta of broilers at the ASV level (based on the Bray–Curtis distance). **(A)** PCoA of ileal microbiota composition. **(B)** PCoA of cecal microbiota composition. **(C)** PCoA of colonic microbiota composition. Each symbol represents five intestinal microbiotas in one treatment, and distance between symbols reflects relative dissimilarities in community structures. NCON is a corn-soybean meal basal diets group. AMP is a 200 mg/kg antibacterial peptide supplemented group. Probio is 200 mg/kg calsporin supplemented group. MMT is 500 mg/kg montmorillonite supplemented group. CHC is 500 mg/kg activated charcoal-herb extractum complex supplemented group.

After denoising, sequences (4,253,546) from the 75 samples (with an average of 52,513 sequences per sample), representing 19,335 amplicon sequence variants (ASVs), were revealed for the subsequent analyses. Parse curves and species accumulation curves showed that the sampling of each group provided sufficient sequences to reflect the diversity and abundance of bacteria (Supplementary Figure 1). α-Diversity used to measure the distribution of species abundances in a given sample can be indicators of community richness (Sobs, Chao 1, and ACE indexes) and diversity (Shannon index) (Ai et al., 2017; Ji et al., 2017). The α-diversity of the microbiota in the ileum, cecum, and colon were analyzed (Figure 2 and Supplementary Figure 2). In the ileum, CHC treatment decreased community abundance represented by lower Sobs, Chao 1, Ace, and Shannon compared with NCON (*p* < 0.05). The positive controls of AMP and MMT also decreased the community abundance represented by lower Sobs than NCON (*p* < 0.05). At this point of decreasing the richness of bacteria, the characteristic of CHC showed more similarity to MMT in that both of them showed similar Sobs, Chao 1, Ace, and Shannon. Shannon index represents the richness and evenness of the bacterial community. The positive control of probiotics (Probio) showed no significant difference from NCON in changing α-diversity of ileal microbiota. There was no statistically significant difference in α-diversity among the five groups in cecum and colon (Supplementary Figure 2). Principal coordinates analysis (PCoA) based on the ASVs showed a clear difference in community composition among groups (NCON, AMP, Probio, MMT, and CHC) in the ileal, cecal, and colonic microbiota of broilers (Figure 3).

**FIGURE 4:**
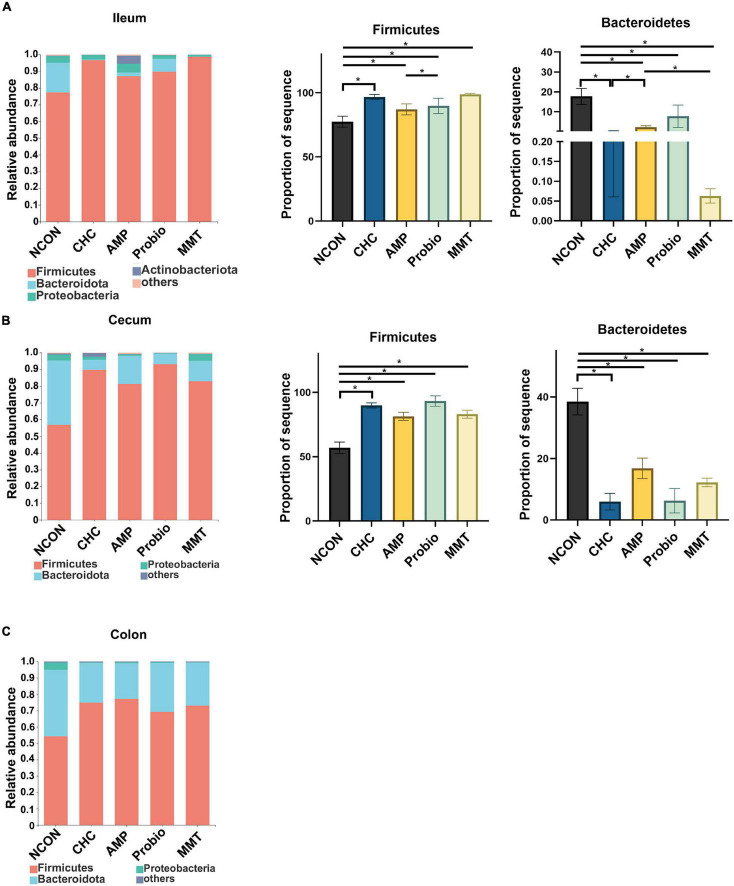
Effects of dietary treatment on ileal, cecal, and colonic microbiota composition at the phylum level. **(A)** Ileal microbiota composition at the phylum level, and alterations of the abundance of bacterial phyla found in the ileum of broilers. **(B)** Cecal microbiota composition at the phylum level, and alterations of the abundance of bacterial phyla found in the cecum of broilers. **(C)** Colonic microbiota composition at the phylum level in broilers. NCON is a corn-soybean meal basal diets group. AMP is 200 mg/kg antibacterial peptide supplemented group. Probio is 200 mg/kg calsporin supplemented group. MMT is 500 mg/kg montmorillonite supplemented group. CHC is 500 mg/kg activated charcoal-herb extractum complex supplemented group. *P* values calculated using the ANCOM test and were adjusted by false discovery rate (FDR) correction according to the Benjamini-Hochberg procedure, and the level of significance was set at FDR < 0.10; * presented FDR < 0.10. Firmicutes (ileum), FDR = 0.031; Bacteroidetes (ileum), FDR = 0.022; Firmicutes (cecum), FDR = 0.014; Bacteroidetes (cecum), FDR = 0.014.

At the phylum level, Firmicutes, Bacteroidetes, and Proteobacteria were predominant phyla in the ileal, cecal, and colonic microbiota in all five groups (Figure 4). In the ileum and cecum, the abundances of Firmicutes in CHC treatment and three positive controls were higher than that of NCON, while the abundances of Bacteroidetes in these groups were lower than that of NCON. In the colon, the abundances of Firmicutes and Bacteroidetes were not differently distributed.

**FIGURE 5:**
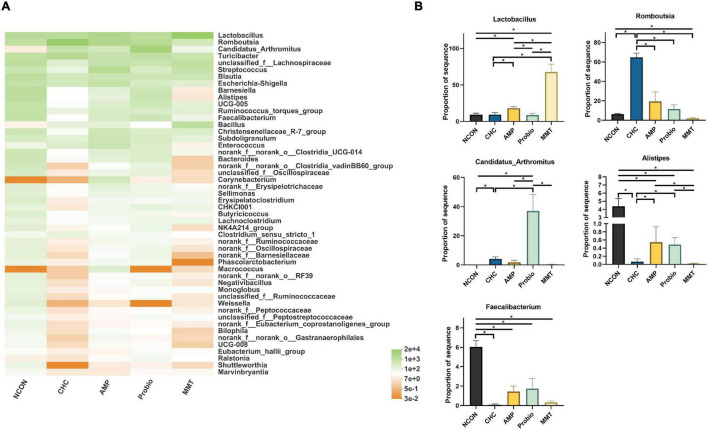
Effects of dietary treatment on intestinal microbiota composition in the ileum at the genus level. **(A)** Ileal bacterial community heatmap at the genus level in broilers. **(B)** Significant alterations of the abundance of bacterial genera found in the ileum of broilers. NCON is a corn-soybean meal basal diets group. AMP is 200 mg/kg antibacterial peptide supplemented group. Probio is 200 mg/kg calsporin supplemented group. MMT is 500 mg/kg montmorillonite supplemented group. CHC is 500 mg/kg activated charcoal-herb extractum complex supplemented group. *P* values calculated using the ANCOM test and were adjusted by false discovery rate (FDR) correction according to the Benjamini-Hochberg procedure, and the level of significance was set at FDR < 0.10; * presented FDR < 0.10. *Lactobacillus*, FDR = 0.060; *Romboutsia*, FDR = 0.061; *Candidatus_Arthromitus*, FDR = 0.077; *Alistipes*, FDR = 0.094; *Faecalibacterium*, FDR = 0.096.

A heatmap exhibited similarities and differences in bacterial communities at the genus level (Figure 5). *Lactobacillus*, *Romboutsia*, and *Candidatus_Arthromitus* were the dominant genera in the ileum, whereas *Lactobacillus*, *Romboutsia*, and *Barnesiella* were the dominant genera in the cecum and colon. The abundances of *Lactobacillales* in the CHC treatment were lower (*p* < 0.05) than those of the AMP and MMT treatments in the ileum. The abundances of *Romboutsia* in the ileum in the CHC group were higher (*p* < 0.05) than those of the three positive controls. The abundances of *Candidatus_Arthromitus* in the ileum in CHC-fed broilers were lower (*p* < 0.05) than those of Probio treatment and higher (*p* < 0.05) than those of NCON treatment. In the ileum, the abundances of *Alistipes* and *Faecalibacterium* in CHC treatment were similar to the three positive controls, which were all lower than that of NCON. CHC treatment had lower abundances of *Alistipes* in the ileum of broilers (*p* < 0.05) relative to those in the AMP and Probio treatments. The absorption effect of CHC on *Candidatus_Arthromitus*, *Alistipes*, and *Faecalibacterium* was similar to that of MMT. In the cecum and colon, there were no significant differences in the abundances of these dominant genera across treatments.

At the genus level, in the ileum compared with NCON, CHC treatment had significantly lower (*p* < 0.05) abundances of the pathogenic bacteria *Alistipes* (Figure 5). The abundance of pathogenic bacteria (*Alistipes*) in CHC treatment was lower (*p* < 0.05) than those of the AMP and Probio treatments. Compared with MMT, CHC treatment had a higher (*p* < 0.05) abundance of *Romboutsia* compared to NCON, AMP, Probio, and MMT. In addition, compared with NCON, CHC had a higher (*p* < 0.05) abundance of *Candidatus_Arthromitus*. However, CHC had no effect on the abundance of *Lactobacillus* which were lactic acid-producing bacteria that were beneficial for gut health. Compared to NCON, Probio treatment had lower (*p* < 0.05) abundances of *Alistipes*; and higher (*p* < 0.05) abundance of *Candidatus_Arthromitus*.

**FIGURE 6:**
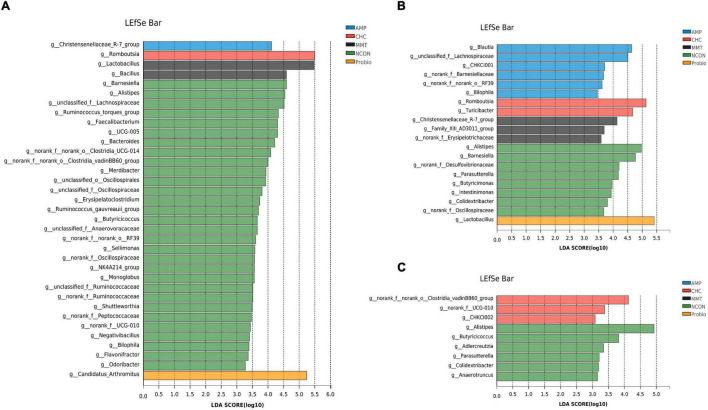
LEfSe analysis shows the most differentially abundant taxa among treatment groups. **(A)** Difference in the abundance of species among five groups in the ileum. **(B)** Difference in abundance of species among five groups in the cecum. **(C)** Difference in abundance of species among five groups in the colon. LDA score > 3.5 is considered to be statistically significant. NCON is a corn-soybean meal basal diets group. AMP is 200 mg/kg antibacterial peptide supplemented group. Probio is 200 mg/kg calsporin supplemented group. MMT is 500 mg/kg montmorillonite supplemented group. CHC is 500 mg/kg activated charcoal-herb extractum complex supplemented group.

Differences in the composition of intestinal microbiota at the genus level were further explored by the LEfSe analysis (Figure 6). In the ileum, we found that 35 bacterial genera including gram-negative bacteria (such as *Barnesiella*, *Alistipes*, *unclassified_f__Lachnospiraceae*) and gram-positive bacteria (such as *UCG-005*, *[Ruminococcus]_torques_group*, *Faecalibacterium*) were enriched in the NCON group. The AMP group was characterized by a higher relative abundance of gram-negative bacteria (*Christensenellaceae_R-7_group*). In addition, gram-positive bacteria (*Romboutsia*), gram-negative bacteria (*Candidatus_Arthromitus*), and gram-positive bacteria (*Lactobacillus* and *Bacillus*) were enriched in CHC, Probio, and MMT groups, respectively.

In the cecum, gram-negative bacteria (*Alistipes*, *Barnesiella*, *norank_f__Desulfovibrionaceae*, *Parasutterella*, *Butyricimonas*, *Intestinimonas*, and *norank_f__Oscillospiraceae*) and gram-positive bacteria (*Colidextribacter* and *Lactobacillus*) in the NCON group had the highest richness among the five treatment groups. We also found gram-positive bacteria (*Blautia*), gram-negative bacteria (*Barnesiellaceae*, *unclassified_f__Lachnospiraceae*, *Bilophila*), and not classified bacteria (*CHKCI001*, *norank_f__norank_o__RF39*, and *Marvinbryantia*) were enriched in the AMP group. Furthermore, gram-positive bacteria (*Romboutsia* and *Turicibacter*) were particularly abundant in response to CHC, while only *Lactobacillus* (gram-positive bacteria) was enriched in the Probio group. The distribution of gram-negative bacteria (*Christensenellaceae_R-7_group*), gram-positive bacteria (*norank_f__Erysipelotrichaceae*), and *Family_XIII_AD3011_group* in group MMT were richer than those in other groups.

In the colon, the NCON group showed enrichment of gram-negative bacteria (*Alistipes*, *Parasutterella*, and *Anaerotruncus*) and gram-positive bacteria (*Butyricicoccus*, *Adlercreutzia*, and *Colidextribacter)*. Furthermore, the CHC group was characterized by a higher relative abundance of gram-positive bacteria (*norank_f__Clostridiales_vadinBB60_group*, *Ruminococcaceae_UCG-010*, and *Senegalimassilia*) and not classified bacteria (*CHKCI002*) than that in other groups.

### Associations Between Intestinal Microbiota, Inflammatory, and Immune Factors

**FIGURE 7:**
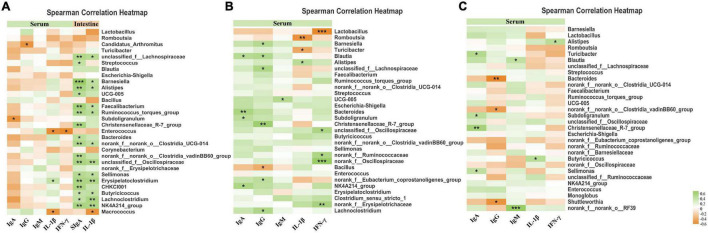
The Spearman correlation among intestinal microbiota, anti-inflammatory, and immune parameters. **(A)** Heatmap of Spearman’ s correlation between ileal microbiota, anti-inflammatory, and immune parameters. **(B)** Heatmap of Spearman’ s correlation between cecal microbiota, anti-inflammatory and immune parameters. **(C)** Heatmap of Spearman’s correlation between Colonic microbiota, anti-inflammatory, and immune parameters. The correlation was analyzed based on the relative abundance of 30 phylotypes at the genus level. Cells are colored based on the correlation coefficient between the significantly altered bacteria and anti-inflammatory or immune parameters. The green suggests a positive correlation, and the yellow suggests a negative correlation. The “*” indicates 0.01 < *p* ≤ 0.05, “**” indicates 0.001 < *p* ≤ 0.01, and “***” indicates *p* ≤ 0.001.

A Spearman correlation analysis was carried out to determine if any relationship existed among serum inflammatory traits, serum immunological markers, and intestinal microbiota (Figure 7).

In the ileum, the levels of serum IgA were negatively associated with *Subdoligranulum* and *Candidatus_Arthromitus* (*p* < 0.05). In addition, the levels of serum IL-1β and TNF-α were positively associated with the abundance of *Erysipelatoclostridium*, while negatively associated with the abundance of *Enterococcus* and *Macrococcus*. Levels of ileum mucosa SIgA and IL-1β were strongly associated with a series of bacteria (e.g., *Unclassified_f__Lachnospiraceae*, *Barnesiella*, *Alistipes*, *Faecalibacterium*, and *Ruminococcus_torques_group*) which indicated an active immune response induced by ileal bacteria.

In the cecum, the immune globulins (higher levels of IgA, IgG, and IgM indicating greater immune functions) exhibited a positive association with the abundance of *Lachnoclostridium*, *NK4A214_group*, *norank_f__Eubacterium_coprostanoligenes_group*, *Christensenellaceae_R-7_group*, *Subdoligranulum*, *Ruminococcaceae_UCG-005*, *Streptococcus*, *unclassified_f__Lachnospiraceae*, *Blautia*, and *Barnesiella*. In addition, correlation analysis revealed that *norank_f__Erysipelotrichaceae*, *norank_f__Oscillospiraceae*, *norank_f__Ruminococcaceae*, *unclassified_f__Oscillospiraceae*, and *Alistipes* had a strong positive association with the level of inflammatory factors (IL-1β and TNF-α) in the serum, while the bacteria *Romboutsia* and *Lactobacillus* had a strong negative association with the serum level of inflammatory factors (IL-1β and TNF-α).

In the colon, the abundance of *norank_f__norank_o__RF39*, *Sellimonas*, *Christensenellaceae_R-7_group*, *Subdoligranulum*, *Blautia*, and *Turicibacter* were positively associated with immune globulins (IgA and IgM), while the abundance of *Shuttleworthia*, *norank_f__Clostridiales_vadinBB60_group*, and *Bacteroides* were negatively associated with the levels of IgG. Lower levels of IL-1β and IFN-γ displayed a negative association with the abundance of *Butyricicoccus* and *Alistipes*.

To better understand the microbiota dynamic changes in broilers, potential functions of microbiota in the ileum, cecum, and colon were predicted by the PICRUSt algorithm (Supplementary Table 1). In the ileum, the functions related to energy production and conversion; transport and metabolism of amino acid, lipid and inorganic ion transport and metabolism; transcription, replication, recombination, and repair; secondary metabolites biosynthesis, transport, and catabolism; and signal transduction mechanisms in CHC group showed an increase (*p* < 0.05) compared with MMT group. There was no difference in the functions in the cecum and colon among treatment groups (*p* > 0.05).

## Discussion

Feed contamination with microbic or toxic agents can deteriorate the structure of the gastrointestinal tract and cause liver injury, which decreases the growth performance of animals. A practical approach is to use mycotoxin absorbents to minimize the negative effect of mycotoxins in feed. Another approach is the use of antibiotics to reduce susceptibility to infectious disease and improve growth performance, which has led to the widespread use of antibiotics in animal feed as growth promoters and medicated feed additives (Chattopadhyay, 2014). However, continuous usage of antibiotics may contribute to the emergence of a reservoir of drug-resistant bacteria, which may then transfer their resistance to pathogenic bacteria in both animals and humans (Bartlett et al., 2013). As a result, much research has focused on the development of antibiotic alternatives to maintain or improve animal performance and health.

Active charcoal is one of these absorbents, which is produced from charcoal in the presence of activation regents. Active charcoal is highly porous and has a high specific surface area that can effectively absorb toxins to physically trap molecules (Brooks et al., 2017; Mohammad et al., 2020). Montmorillonite (MMT) is another example of clay absorbents that are beneficial to broiler growth performance, feed efficiency, and gut morphology (Ma and Guo, 2008) due to its absorption capacity of feed mycotoxins (Abbès et al., 2012; Yang et al., 2014; Saleemi et al., 2020). Loading MMT with copper (Ma and Guo, 2008), calcium (Lillehoj et al., 2016), zinc oxide (Hu et al., 2013), or antibiotics (Qu et al., 2014) imparts additional functions to MMT from these ligands such as increasing the activities of endogenous digestive enzymes, improving intestinal morphology and acting as an antibacterial agent. Modification of active charcoal with antibacterial agents can enhance the activity of the antibacterial by up to 72% compared with the application of pure antibiotics (Burchacka et al., 2021).

Chinese herbal medicine resources are rich in China. Chinese herbal medicine additives contain a variety of trace elements and bioactive compounds, with antibacterial, anticancer, and anti-inflammatory properties (Guo, 2021). Chinese herbals have been widely used in human medicine, animal feed, food, and cosmetic industries (Lyu et al., 2017; Luo et al., 2019; Shi, 2019; Shen, 2021). Resources of some Chinese herbs like *P. chinensis*, *P. oleracea L.*, *A. argyi Folium*, and *P. multifida Poir* are rich because of easy growing independent of areas, temperature, and humidity. Activated charcoal was a highly porous form of carbon that could trap chemicals efficiently (Brooks et al., 2017; Mohammad et al., 2020). CHC which couples active charcoal with Chinese herbs (*P. chinensis*, *P. oleracea L.*, *A. argyi Folium*, and *P. multifida Poir*) may have dual functions in both absorptions like clays and antibacterial activity. Compared with MMT, CHC had 30-fold more surface area, seven-fold greater absorptive capacity, and five-fold greater pore distribution. A previous *in vitro* study revealed that CHC was able to absorb mycotoxins including deoxynivalenol, zearalenone, and fumonisin B1 (Wang et al., 2019).

In the current study in broilers, CHC supplementation (optimal dose of 500 mg/kg CHC), improved growth performance and tended to improve feed conversion rate. To reveal CHC functional mechanisms, three positive controls were selected: AMP prebiotics, Probio (*Bacillus subtilis*) representing probiotics, and MMT representing absorbents. Results showed that the treatments of three positive controls had no significant difference from NCON on growth performance. One of the reasons was the treatment doses of these positive controls were not optimized. Environmental challenges like pathogens and temperatures can also contribute to the various effects of prebiotics and probiotics on broiler growth performance (Santoso et al., 1999; Xia et al., 2004; Biggs et al., 2007; Sohail et al., 2012; Abudabos et al., 2020).

Charcoal-herb extractum complex improved both cellular immune and humoral immune responses as indicated by serum IL-1β, IFN-γ, and IgA concentrations. IL-1β and IFN-γ are important inflammatory cytokines that play crucial roles in the pathogenesis of a range of inflammatory and autoimmune diseases (Rožman and Švajger, 2018; Cheng et al., 2019). The observed stimulation of immune responses was attributed to the ligands of Chinese herbs. Chinese herbs contain numerous active compounds related to anti-inflammatory and antibacterial functions (Guan et al., 2006; Liu et al., 2013; Iranshahy et al., 2017; Cao et al., 2020). Another important role of CHC was to modulate bacterial composition in the intestinal tract. First, CHC, like other positive control treatments (AMP, Probio, and Absorb), lowered the diversity of species abundance in the ileum. Decreasing the richness of bacteria caused by CHC was similar to responses attributable to MMT (MMT). In the cecum and colon, CHC and MMT showed no significant difference, which indicated that the absorptive functions of CHC and MMT acted in the foregut rather than the hindgut. Second, CHC inhibited the abundance of *Alistipes* which is harmful bacteria related to causing intestinal inflammation in the ileum (Schirmer et al., 2019; Valido et al., 2022). This result suggested that although CHC had advantages in surface area and pore size, the function of absorption may also rely on other properties such as hydrophobic character, distances between porous sheets, and electrical conductivity (Nadziakiewicza et al., 2019).

Charcoal-herb extractum complex had no beneficial effect on the abundance of *Lactobacillus*, which was the lactic acid-producing bacteria. These characteristics of CHC on the abundance of specific pathogenic bacteria were likely represented by the composition of Chinese herbs. In other words, changing the composition of extractum of Chinese herbs may change the inhibition or production of specific bacteria. Compared with CHC, MMT favored the abundance of *Lactobacillus* in the ileum. MMT was beneficial to the survival of strains in the gastrointestinal tract (Nadziakiewicza et al., 2019), and combination administration of *Lactobacillus* with MMT protected rats from mytoxin-induced immune disorders (Ben Salah-Abbès et al., 2016). Compared with CHC, AMP, and MMT, interestingly, the addition of Probio (*Subtilis bacillus*) did not cause significant alteration of *Lactobacillus* abundance in the gastrointestinal tract. However, the addition of Probio decreased the abundance of harmful bacteria *Alistipes*, and enhanced the abundance of beneficial bacteria *Candidatus_Arthromitus*. This indicated that as with other probiotics, *S. bacillus* expressed antibacterial properties by producing bacteriostatic substances such as organic acids, bacteriocins, and antibiotics (Rolfe, 2000).

Charcoal-herb extractum complex increased the abundance of Firmicutes and decreased Bacteroidetes in the ileum and cecum. Firmicutes were dominant in the ileum, cecum, and cecum of broilers (Rychlik, 2020). Firmicutes play a crucial role in host health, including regulating host immunity and maintaining intestinal barrier integrity (Louis and Flint, 2017). Meanwhile, Bacteroidetes have the ability to release LPS, which then leads to heightened inflammatory responses (Ortega-Hernández et al., 2020). Thus, a decreased proportion of Bacteroidetes is related to lower inflammatory factors. A higher Firmicutes/Bacteroidetes ratio is associated with enhanced lower inflammation and reduced infection risk in humans (Mariat et al., 2009) and pigs (Molist et al., 2012; Hu et al., 2020). Therefore, we speculate that CHC supplementation exhibits immune and anti-inflammatory effects closely associated with the increased abundance of Firmicutes and decreased abundance of Bacteroidetes in ileum and cecum of broilers.

We also conducted a correlation analysis on immune responses and intestinal bacteria. In the cecum, the abundance of *Romboutsia* was associated negatively with serum levels of IL-1β. *Lactobacillus* abundance was associated negatively with IFN-γ in the cecum. IL-1β is secreted by the sentinel cells (macrophage and monocytes) of the innate immune system (Granowitz et al., 1992) or other cell types like epithelial, endothelial, and fibroblasts (Hoffmann et al., 2005; Choi et al., 2008). IFN-γ is produced by NK cells, T and B cells (Harris et al., 2000). These negative associations suggest that *Romboutsia*, and *Lactobacillus* promote positive immune responses. *Lactobacillus* strains were proved to alleviate inflammation by inhibition of IL-1β expression (Yun et al., 2020) *in vivo*, and activated macrophages are multi-faceted *in vitro* (Rocha-Ramírez et al., 2017). *Romboutsia* was important bacteria that was associated with the health status of the gastrointestinal tract (Ricaboni et al., 2016). The drastic reduction of *Romboutsia* in intestinal mucosa may represent a potential microbial indicator of intestinal dysbiosis (Mangifesta et al., 2018).

We also found a strong positive association between a series of bacteria and ileum mucosal SIgA and ileum mucosal IL-1β. SIgA is involved in the mucosal immune response that can protect against various pathogens by neutralization, inhibition of adherence, and agglutination. SIgA does not activate the complement pathway, thus, it is more anti- than proinflammatory (Russell et al., 2015). *Alistipes* were highly relevant in diseases, such as liver fibrosis (Rau et al., 2018), colorectal cancer (Moschen et al., 2016), cardiovascular disease (Zuo et al., 2019), and mood disorders (Bangsgaard Bendtsen et al., 2012). *Faecalibacterium* in the intestine was significantly enriched in patients with tuberculosis (Maji et al., 2018) or Crohn’s disease (Hansen et al., 2012). These results indicated that the bacteria like *Alistipes* and *Faecalibacterium* could locally stimulate the secretion of inflammatory factors by intestinal cells, and increase mucosal immune response.

## Conclusion

In summary, CHC supplementation (optimal dose of 500 mg/kg) increased growth performance and tended to increase feed conversion rate in broilers. It had a beneficial effect on intestinal microbial composition and enhanced the immune status of broilers. CHC supplementation increased the abundance of Firmicutes in the ileum and cecum, while decreasing the abundance of Bacteroidetes in ileum and cecum. CHC markedly reduced the abundances of pathogenic bacteria *Alistipes*, while enriching the abundances of beneficial bacteria (*Romboutsia* and *Lactobacillus*) in the ileum. The bacteria (*Faecalibacterium* and *Alistipes*) were strongly negatively associated with the levels of mucosa SIgA and IL-1β in the ileum. CHC decreased the level of the inflammatory cytokines IL-1β and IFN-γ in serum, and improved immune status (represented by the levels of IgA and IgM in serum, and SIgA in the mucosa of duodenum and jejunum). CHC demonstrated dual functions of absorption like clay and antibacterial like antibacterial peptides.

## Data Availability Statement

The datasets presented in this study can be found in online repositories. The names of the repository/repositories and accession number(s) can be found in the article/Supplementary Material.

## Ethics Statement

The animal study was reviewed and approved by the Laboratory Animal Welfare and Animal Experimental Ethical Inspection Committee of China Agricultural University. Written informed consent was obtained from the owners for the participation of their animals in this study.

## Author Contributions

LW and BD designed and wrote the manuscript. YZ and XG critically edited the text. LW, LG, and BD finalized the manuscript. All authors read and approved the final manuscript.

## Conflict of Interest

The authors declare that the research was conducted in the absence of any commercial or financial relationships that could be construed as a potential conflict of interest.

## Publisher’s Note

All claims expressed in this article are solely those of the authors and do not necessarily represent those of their affiliated organizations, or those of the publisher, the editors and the reviewers. Any product that may be evaluated in this article, or claim that may be made by its manufacturer, is not guaranteed or endorsed by the publisher.

## Supplementary Material

Supplementary Figure 1 Dilution curve and rank abundance curve. **(A)** Rarefaction curves for total amplicon sequence variants (ASV), showing the sequencing depth with increasing sequencing depth. **(B)** Rank Abundance curve, showing the richness and evenness of the observed species (97% identity) based on the 16S rDNA gene.

Supplementary Figure 2 Effects of CHC on α-diversity of cecal and colonic microbiota in broilers on day 42. Bar graphs show changes in indexes of **(A)** Sobs (upper left panel), Chao 1 (upper right panel), ACE (bottom left panel), and Shannon (bottom right panel) in cecum, **(B)** Sobs (upper left panel), Chao 1 (upper right panel), ACE (bottom left panel), and Shannon (bottom right panel) in colon. Four indexes analyzed the α diversity of broilers from different directions. NCON is a corn-soybean meal basal diets group. AMP is 200 mg/kg antibacterial peptide supplemented group. Probio is 200 mg/kg calsporin supplemented group. MMT is 500 mg/kg montmorillonite supplemented group. CHC is 500 mg/kg activated charcoal-herb extractum complex supplemented group.

Supplementary Figure 3 Effects of dietary treatment on intestinal microbiota composition at the genus level in the cecum and colon. **(A)** Cecal bacterial community heatmap in broilers. **(B)** Colonic bacterial community heatmap in broilers. NCON is a corn-soybean meal basal diets group. AMP is 200 mg/kg antibacterial peptide supplemented group. Probio is 200 mg/kg calsporin supplemented group. MMT is 500 mg/kg montmorillonite supplemented group. CHC is 500 mg/kg activated charcoal-herb extractum complex supplemented group.
