# Supplementary figures and images for: Beneficial Alteration in Growth Performance, Immune Status, and Intestinal Microbiota by Supplementation of Activated Charcoal-Herb Extractum Complex in Broilers
Source: Front Microbiol. 2022 Apr 15;13:856634. doi: 10.3389/fmicb.2022.856634 (PMC9051449; doi:10.3389/fmicb.2022.856634)

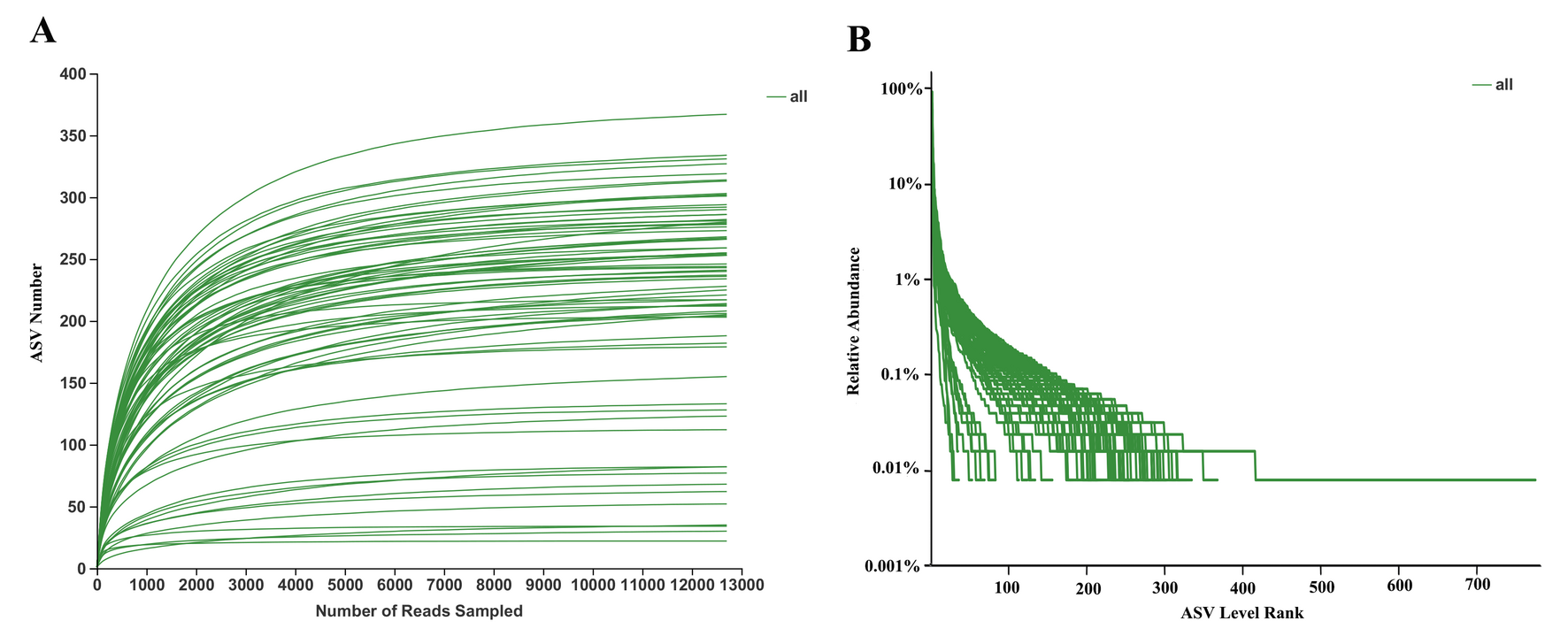

Supplement: Supplementary Figure 1 — Dilution curve and rank abundance curve. (A) Rarefaction curves for total amplicon sequence variants (ASV), showing the sequencing depth with increasing sequencing depth. (B) Rank Abundance curve, showing the richness and evenness of the observed species (97% identity) based on the 16S rDNA gene. [file Image_1.TIF]

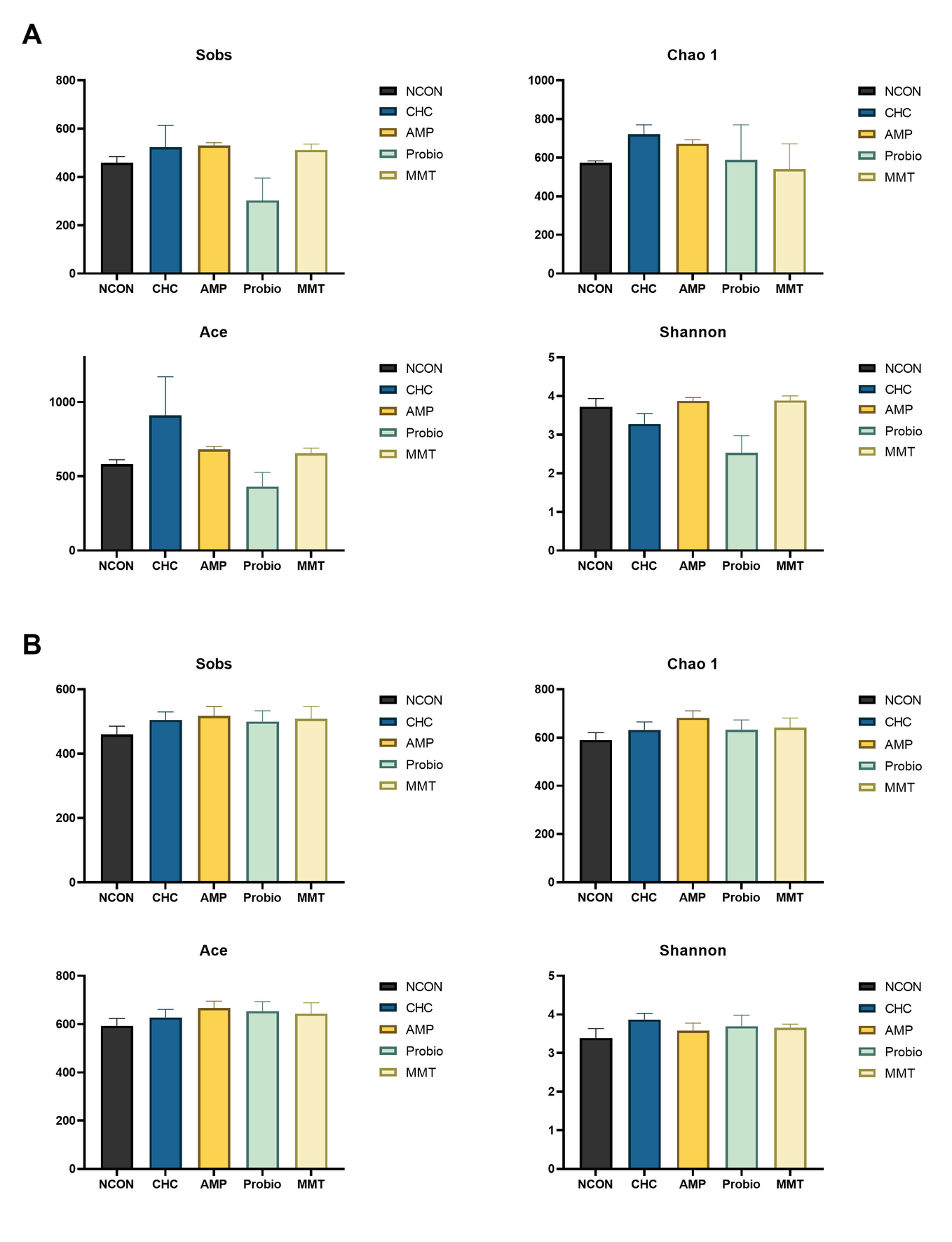

Supplement: Supplementary Figure 2 — Effects of CHC on α-diversity of cecal and colonic microbiota in broilers on day 42. Bar graphs show changes in indexes of (A) Sobs (upper left panel), Chao 1 (upper right panel), ACE (bottom left panel), and Shannon (bottom right panel) in cecum, (B) Sobs (upper left panel), Chao 1 (upper right panel), ACE (bottom left panel), and Shannon (bottom right panel) in colon. Four indexes analyzed the α diversity of broilers from different directions. NCON is a corn-soybean meal basal diets group. AMP is 200 mg/kg antibacterial peptide supplemented group. Probio is 200 mg/kg calsporin supplemented group. MMT is 500 mg/kg montmorillonite supplemented group. CHC is 500 mg/kg activated charcoal-herb extractum complex supplemented group. [file Image_2.TIF]

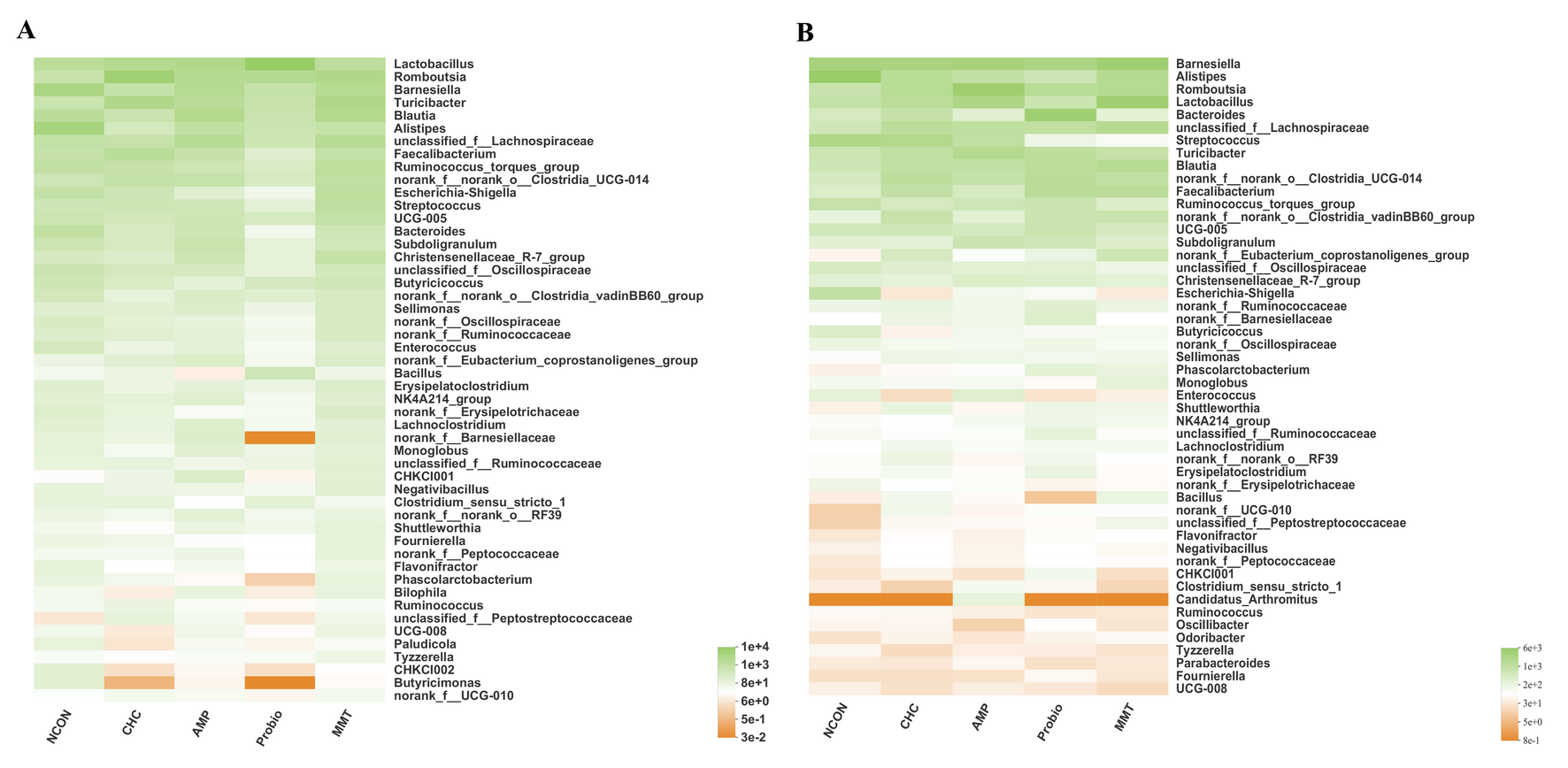

Supplement: Supplementary Figure 3 — Effects of dietary treatment on intestinal microbiota composition at the genus level in the cecum and colon. (A) Cecal bacterial community heatmap in broilers. (B) Colonic bacterial community heatmap in broilers. NCON is a corn-soybean meal basal diets group. AMP is 200 mg/kg antibacterial peptide supplemented group. Probio is 200 mg/kg calsporin supplemented group. MMT is 500 mg/kg montmorillonite supplemented group. CHC is 500 mg/kg activated charcoal-herb extractum complex supplemented group. [file Image_3.TIF]
