# Supplementary material for: Beneficial Alteration in Growth Performance, Immune Status, and Intestinal Microbiota by Supplementation of Activated Charcoal-Herb Extractum Complex in Broilers
Source: Front Microbiol. 2022 Apr 15;13:856634. doi: 10.3389/fmicb.2022.856634 (PMC9051449; doi:10.3389/fmicb.2022.856634)
Supplement: Supplementary file 4 [file Table_1.DOCX]

**Supplemental TABLE 1** Predicted metabolism of intestinal microbiota using PICRUSt analysis. ^1^

| **Items** | **NCON** | **CHC** | **AMP** | **Probio** | **MMT** | **SEM** | ***P* value** |
| --- | --- | --- | --- | --- | --- | --- | --- |
| **Ileum** | | | | | | | |
| Energy production and conversion | 923351.6^a^ | 522093.6^b^ | 773150.1^a^ | 555117.6^b^ | 761863.2^a^ | 39009.542 | <0.01 |
| Amino acid transport and metabolism | 1526254.7^a^ | 827061.2^b^ | 1437838.1^a^ | 923382.4^b^ | 1503952.3^a^ | 74011.087 | < 0.01 |
| Nucleotide transport and metabolism | 617700.1^b^ | 370961.8^c^ | 613103.0^b^ | 401683.9^c^ | 803470.8^a^ | 35379.849 | <0.01 |
| Carbohydrate transport and metabolism | 1217403^a^ | 645648.7^b^ | 1191434.9^a^ | 771633.2^b^ | 1408213.6^a^ | 67230.215 | <0.01 |
| Coenzyme transport and metabolism | 569055.9^a^ | 326838.3^b^ | 489499.4^a^ | 334730.0^b^ | 463372.0^a^ | 23618.425 | <0.01 |
| Lipid transport and metabolism | 383340.5^b^ | 219574.0^c^ | 370671.8^b^ | 251235.8^c^ | 452151.1^a^ | 19865.42 | <0.01 |
| Translation, ribosomal structure, and biogenesis | 1458105.4^b^ | 844148.2^c^ | 1368376.0^b^ | 978768.7^c^ | 1713795.6^a^ | 72770.624 | <0.01 |
| Transcription | 1290040.6^a^ | 724222.6^b^ | 1153183.5^a^ | 788075.2^b^ | 1188711.3^a^ | 59833.56005 | <0.01 |
| Replication, recombination, and repair | 1051305.1^b^ | 573297.6^c^ | 972853.6^c^ | 670034.0^c^ | 1232317.8^a^ | 55470.403 | <0.01 |
| Inorganic ion transport and metabolism | 910906.6^a^ | 601255.8^b^ | 875101.6^a^ | 598913.1^b^ | 823495.3^a^ | 34606.504 | <0.01 |
| Secondary metabolites biosynthesis, transport, and catabolism | 70022.1^a^ | 40585.8^b^ | 73272.6^a^ | 46103.9^b^ | 56666.7^ab^ | 3695.98 | <0.01 |
| Signal transduction mechanisms | 588911.3^a^ | 335043.4^b^ | 489344.1^a^ | 360289.4^b^ | 487730.2^a^ | 24781.926 | <0.01 |
| **Cecum** | | | | | | | |
| Energy production and conversion | 903733.2^a^ | 739451.3^b^ | 883815.0^a^ | 727895.5^b^ | 909508.4^a^ | 24266.632 | 0.01 |
| Amino acid transport and metabolism | 1435605.1^a^ | 1229604.6^b^ | 1466689.6^a^ | 1352331^ab^ | 1514679.4^a^ | 33713.11 | 0.048 |
| Nucleotide transport and metabolism | 576746.3^bc^ | 522163.1^c^ | 612343.4^b^ | 700706.9^a^ | 611441.6^b^ | 16383.21 | <0.01 |
| Carbohydrate transport and metabolism | 1184793.2^a^ | 939157.8^b^ | 1215550.5^a^ | 1212021.2^a^ | 1206581.8^a^ | 31211.092 | <0.01 |
| Coenzyme transport and metabolism | 543991.9^a^ | 451627.1^b^ | 541379.5^a^ | 418579.1^b^ | 559510.4^a^ | 16110.306 | <0.01 |
| Lipid transport and metabolism | 371916.5^a^ | 317775.6^b^ | 365014.4^a^ | 389736.7^a^ | 374905.1^a^ | 7834.568 | 0.029 |
| Translation, ribosomal structure, and biogenesis | 1344944.8^ab^ | 1212803.0^b^ | 1432141.4^a^ | 1514337.5^a^ | 1458990.7^a^ | 33366.657 | 0.024 |
| Transcription | 1162359.1^abc^ | 1053755.1^c^ | 1264395.7^ab^ | 1094611.1^bc^ | 1311766.2^a^ | 33370.615 | 0.054 |
| Replication, recombination, and repair | 987181.4^ab^ | 858730.1^b^ | 1018856.9^a^ | 1075322.9^a^ | 1041278.4^a^ | 25599.524 | 0.058 |
| Inorganic ion transport and metabolism | 873910.8^ab^ | 780138.4^bc^ | 885034.3^ab^ | 746581.8^c^ | 926202.6^a^ | 20919.307 | 0.017 |
| Secondary metabolites biosynthesis, transport, and catabolism | 70595 | 61665.5 | 61189.7 | 56742.4 | 65139.5 | 2009.861 | 0.268 |
| Signal transduction mechanisms | 550239.2^ab^ | 468975.4^b^ | 574187.6^a^ | 468351.7^b^ | 584483.5^a^ | 16138.804 | 0.026 |
| **Colon** | | | | | | | |
| Energy production and conversion | 819294.4^b^ | 884614.7^ab^ | 767282.4^b^ | 962794.5^a^ | 795797.3^b^ | 21562.152 | 0.016 |
| Amino acid transport and metabolism | 1378726.2 | 1504140.8 | 1288446.8 | 1538109.6 | 1330355.9 | 34973.842 | 0.086 |
| Nucleotide transport and metabolism | 552676.9 | 608234.9 | 545848 | 621798.8 | 572852.6 | 13746.742 | 0.326 |
| Carbohydrate transport and metabolism | 1128042.4 | 1184569.9 | 1026592.2 | 1191103.1 | 1071907.2 | 29826.828 | 0.351 |
| Coenzyme transport and metabolism | 503171.4^b^ | 546291.8^ab^ | 469014.8^b^ | 595199.3^a^ | 484347.0^b^ | 14396.549 | 0.022 |
| Lipid transport and metabolism | 359698.5 | 377466 | 328584.2 | 387278.7 | 344938.5 | 8337.514 | 0.157 |
| Translation, ribosomal structure, and biogenesis | 1289468 | 1438788.1 | 1277487.1 | 1474236.4 | 1329253.6 | 31012.358 | 0.147 |
| Transcription | 1072532.1 | 1246968.9 | 1086873.5 | 1242951.4 | 1109451.8 | 31971.623 | 0.215 |
| Replication, recombination, and repair | 913150 | 1016889 | 898986.2 | 1057908.6 | 941187.6 | 22586.837 | 0.101 |
| Inorganic ion transport and metabolism | 818933.2^b^ | 892549.1^ab^ | 784633.9^b^ | 952570.6^a^ | 773368.1^b^ | 21952.545 | 0.029 |
| Secondary metabolites biosynthesis, transport, and catabolism | 67253.3^ab^ | 66988.0^ab^ | 57063.9^b^ | 76966.6^a^ | 57112.0^b^ | 2422.984 | 0.033 |
| Signal transduction mechanisms | 494064.9^b^ | 565368.2^ab^ | 484564.4^b^ | 603788.8^a^ | 509893.7^b^ | 14700.891 | 0.029 |

^1^ NCON is a corn-soybean meal basal diets group. AMP is 200 mg/kg antibacterial peptide supplemented group. Probio is 200 mg/kg calsporin supplemented group. MMT is 500 mg/kg montmorillonite supplemented group. CHC is 500 mg/kg activated charcoal-herb extractum complex supplemented group. Values were expressed for means ± standard error of the mean (SEM). Means within rows with different letter superscripts differ significantly (*P* < 0.05).
